# Feasibility Study of an Automated Assembly Process for Ultrathin Chips

**DOI:** 10.3390/mi11070654

**Published:** 2020-06-30

**Authors:** Florian Janek, Ebru Saller, Ernst Müller, Thomas Meißner, Sascha Weser, Maximilian Barth, Wolfgang Eberhardt, André Zimmermann

**Affiliations:** 1 Hahn-Schickard, Allmandring 9b, 70569 Stuttgart, Germany; E.Saller@pilz.de (E.S.); Thomas.meissner@balluff.de (T.M.); Sascha.Weser@hahn-schickard.de (S.W.); Maximilian.Barth@hahn-schickard.de (M.B.); Wolfgang.Eberhardt@hahn-schickard.de (W.E.); zimmermann@ifm.uni-stuttgart.de (A.Z.); 2 Institute for Micro Integration (IFM), University of Stuttgart, Allmandring 9B, 70569 Stuttgart, Germany; mueller@ifm.uni-stuttgart.de

**Keywords:** system-in-foil, ultrathin chips, automated assembly

## Abstract

This paper presents a feasibility study of an automated pick-and-place process for ultrathin chips on a standard automatic assembly machine. So far, scientific research about automated assembly of ultrathin chips, with thicknesses less than 50 µm, is missing, but is necessary for cost-effective, high-quantity production of system-in-foil for applications in narrow spaces or flexible smart health systems applied in biomedical applications. Novel pick-and-place tools for ultrathin chip handling were fabricated and a process for chip detachment from thermal release foil was developed. On this basis, an adhesive bonding process for ultrathin chips with 30 µm thickness was developed and transferred to an automatic assembly machine. Multiple ultrathin chips aligned to each other were automatically placed and transferred onto glass and polyimide foil with a relative placement accuracy of ±25 µm.

## 1. Introduction

The challenge for a higher functional diversification in future systems (More-than-Moore) is expected to become more important than the continued scaling of the transistor density (More Moore) [1]. This can be encountered by heterogeneous integration of components with specific tasks, e.g., power control, radio frequency (RF) communication, passive components, sensors, and actuators, into a system-in-package (SiP). This is already an established strategy for rigid system carriers, but still in development for system-in-foil (SiF) applications that feature minimum thicknesses, low weight, and mechanical flexibility. Conventional silicon chips are stiff and brittle. A key component for SiF is the process of silicon chip thinning to achieve ultrathin, mechanically flexible silicon chips. The thinner the chip, the lower the possible bending radius [2]. Various groups published methods to achieve chip thicknesses between 10 µm and 50 µm and investigated their fracture strength after dicing [3,4,5,6,7,8]. The next step aiming towards high-quantity production of SiF is the automated assembly of ultrathin chips on flexible foil substrates. 

After silicon thinning, the chips are detached from the wafer release foil and assembled either by face-down flip-chip technology [9,10,11] or face-up die-bonding with subsequent electrical contacting [12,13,14,15]. There are various disadvantages of flip-chip assembly in regard to ultrathin chips. The handling of ultrathin chips is more challenging than the handling of stiff, standard components because of the warpage of the thinned chips [16,17,18]. The handling of bumped, ultrathin chips complicates the handling process further. In general, the use of isotropic conductive adhesives for bumps and circuitry contacting limits the achievable minimum pitch because of increasing risk of shorting adjacent contacts. On the other hand, particles in anisotropic conductive adhesive can induce cracks in the thinned material [10]. In regard to those disadvantages, a face-up assembly approach was chosen in this feasibility study. Additionally, there are more possibilities for electrical connection after face-up assembly, for example, printing technologies [2], wire bonding [19], and physical vapor deposition PVD sputter processes after surface masking [20,21] with the possibility for additional plating [22]. The interconnection of multiple face-up assembled chips, e.g., by using lithographic processes for small feature sizes or by using printing technologies for adaptive layouts, offers the potential of increased functional diversity for SiP-SiF.

So far, the face-up assembly of ultrathin chips is only performed with single chips [23,24,25,26,27]. Reasons may be the challenging handling of ultrathin chips or difficulties in precise placing of ultrathin components on adhesives and their curing. Only if those aspects can be controlled for the entire process chain can a complex system consisting of multiple ultrathin components be assembled. Besides the missing knowledge about multiple component assembly, there is also no scientific research available about an automated face-up assembly of two or more ultrathin chips using automated assembly machines. This work demonstrates the feasibility of automated face-up assembly of multiple chips with thicknesses of 30 µm assembled on glass and foil substrates. We investigated each single process step, beginning from detaching ultrathin chips from thermal release foil to the construction of suitable detaching and handling tools for ultrathin components until the final transfer to an automated assembly machine for high placement accuracy. 

## 2. Materials and Methods 

### 2.1. Development of a Manual Pick-and-Place Process for Ultrathin Chips 

**Figure 1:**
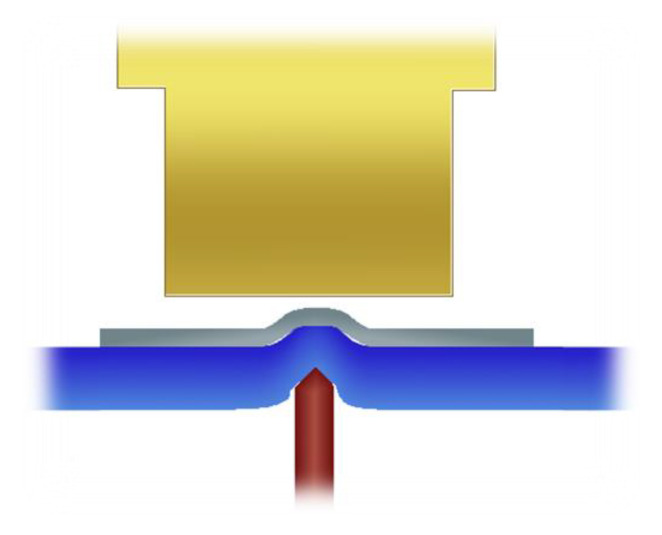
Ultrathin chip on wafer dicing tape. A die ejector tool formed as a needle punctures from below through the wafer dicing tape on the bottom of the chip. Simultaneously, a vacuum tool picks up the chip from above.

In comparison to components with standard thicknesses of more than 400 µm, ultrathin chips should not be detached from wafer dicing tapes with standard die-ejector tools. These tools are shaped as needles or cones and pierce from below the wafer dicing tape on the bottom of the chip, stretching the tape in the area of the chip and weakening the adhesion forces to the chip. Simultaneously, a vacuum tool picks up the chip from above. This procedure often destroys the ultrathin chips due to mechanical stress peaks. The needle rather punctures locally the thinned silicon material while most of the area adheres to the wafer dicing tape (Figure 1). An analytical evaluation of crack formation in ultrathin silicon dies due to the usage of die-ejector tools can be found in [28].

**Figure 2:**
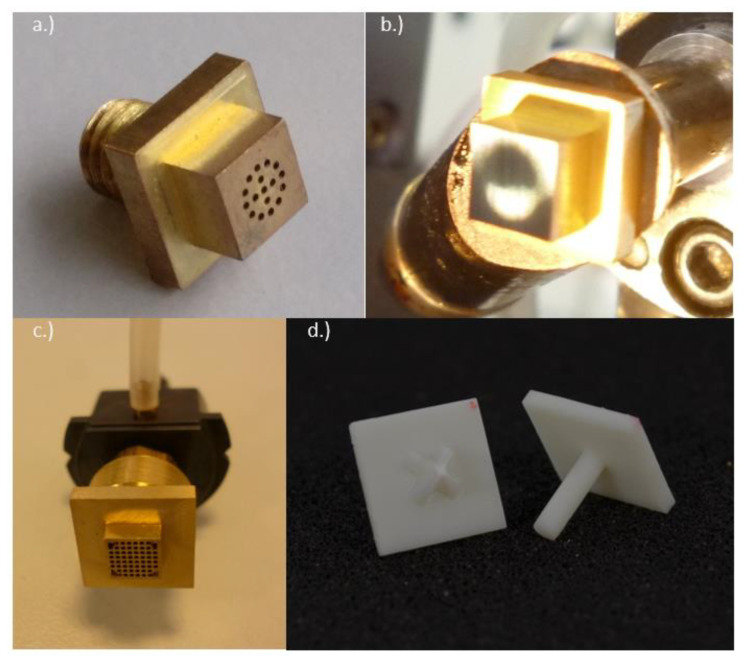
(**a**) Metal tool for ultrathin chip handling with circular vacuum area. (**b**) Bare chip picked with the handle tool shown in (**a**). An inhomogeneous application of vacuum can lead to warpage of the thinned chips. (**c**) Metal handling tool with homogenous vacuum area for automated pick-and-place processes. The tool is integrated into a black-colored mounting adapter needed for the integration into the assembly machine. (**d**) Stamp tool made by an additive manufacturing process called “digital light processing” (DLP). A cross structure enables a repeatable volume during the automated dip and stamp epoxy adhesive transfer.

For this reason, special handling tools for a pick-and-place process for ultrathin chips have been manufactured and investigated to avoid damages. Chip detachment tests were conducted with ultrathin thin chips with 30 µm thickness and lateral dimensions of 4.7 mm × 4.7 mm on thermal release foil (Revalpha Tape 3196, Nitto Denko, Osaka, Japan) and a custom-made, heatable metal handling tool (Figure 2a) on a manual assembly machine (Finetech lambda, Berlin, Germany). The ultrathin chips were detached starting at a temperature of 120 °C. The temperature was increased until no further reduction of detachment time could be achieved. An inhomogeneous vacuum area leads to warpage of the ultrathin chips while being handled (Figure 2b). Thus, metal handling tools with a uniform distribution of vacuum channels have been manufactured (Figure 2c). The vacuum area of the tool is designed smaller (4.5 mm × 4.5 mm) than the used chip size (4.7 mm × 4.7 mm) to avoid contamination of the chip surface with adhesives by capillary forces [29]. 

At first, single, detached chips were placed on dispensed droplets of epoxy-based two-component adhesive (EPO-TEK 354, Epoxy Technology, Billerica, MA, USA) on stiff glass as well as on mechanically flexible polyimide foil with 75 µm thickness (Flexiso PI FI 16000, Dr. Dietrich Müller GmbH, Ahlhorn, Germany). The two-component adhesive was chosen because it is nontoxic, exhibits a viscosity in a practicable viscosity range of 4–6 Pa·s and a comparatively long potlife of three days after mixing the two components. The adhesive was cured in an oven for 10 min at 150 °C. The single, ultrathin chips were successfully bonded onto glass and flexible polyimide foil without damages. 

### 2.2. Transfer to an Automatic Assembly Machine 

**Figure 3:**
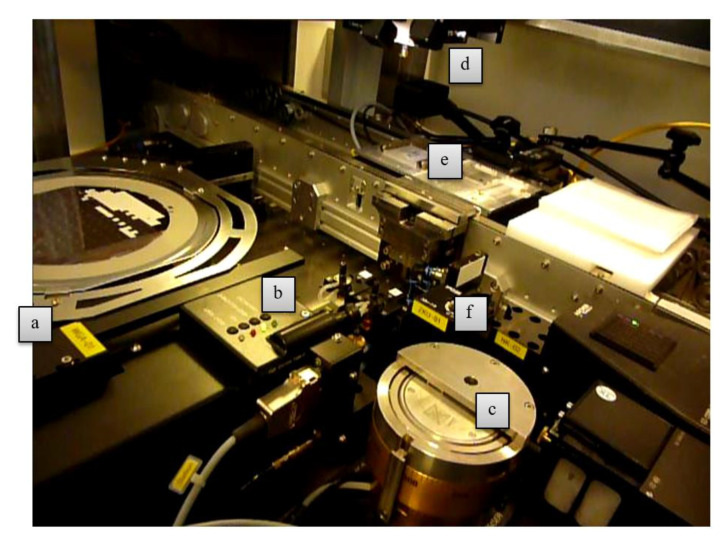
Overview of the assembly machine. (**a**) Wafer feeder, (**b**) tool change station, (**c**) adhesive reservoir, (**d**) head equipped with a tool, (**e**) substrate carrier and cooling plate, (**f**) bottom camera for optical control of lateral and rotational offset of chips on the placement tool.

**Figure 4:**
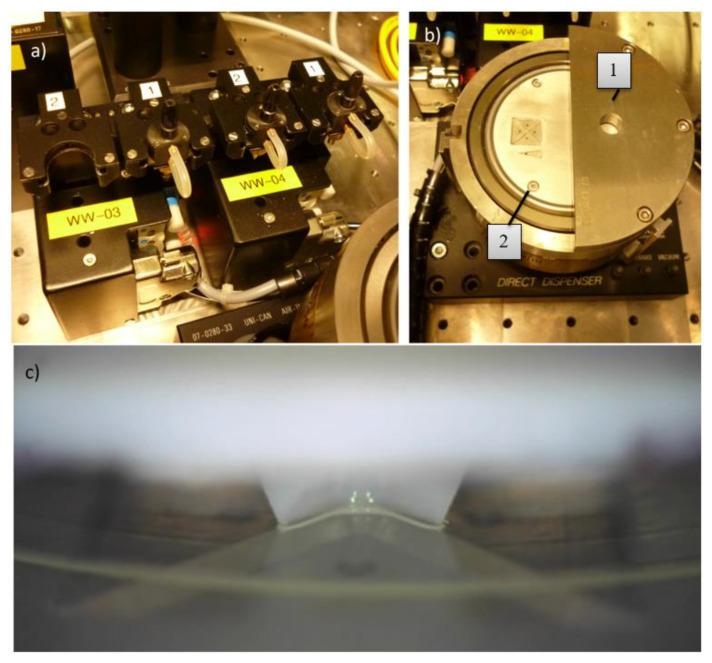
(**a**) Tool change station. Custom-made tools are integrated into a black-colored adapter and stored in mountings. (**b**) Adhesive reservoir module. Adhesive is filled into a circular opening (1). A circular mounting (2) with a stretched foil rotates above a metal disk. The adhesive filled into the opening flows onto the stretched foil. The rotational movement of the circular mounting distributes adhesives evenly on the stretched foil. (**c**) For adhesive transfer, the stamp tool is dipped into the adhesive on the stretched foil.

**Figure 5:**
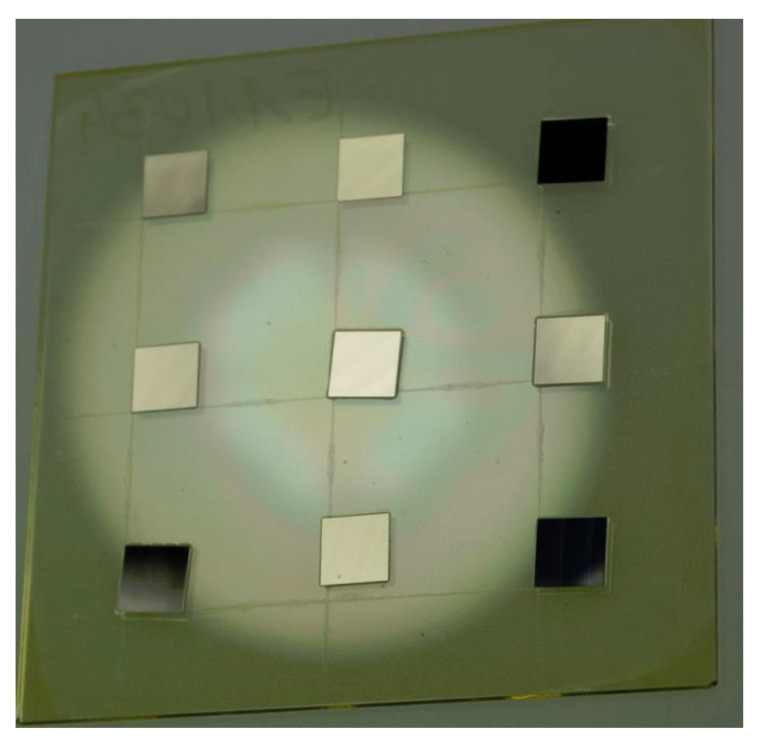
Ultrathin silicon chips placed on liquid epoxy adhesive on glass substrate floating during oven curing, due to missing fixation.

An automatic assembly machine (Type Vico XTec, Haecker Automation GmbH, Waltershausen, Germany, Figure 3) was equipped with two metal tools (Figure 2c) and an additively manufactured stamping tool for epoxy adhesive transfer (Figure 2d). One metal tool was used as heated detachment tool and the other metal tool was used as placement tool. A wafer with ultrathin chips on thermal release foil was loaded into the wafer feeder (Figure 3a) and a substrate was placed onto the substrate carrier (Figure 3e). The equipped stamping tool was checked for correct mounting by image recognition using a bottom camera. After optical check, the stamp was dipped into epoxy adhesive (EPO-TEK 354, Epoxy Technology, Billerica, MA, USA, Figure 4) and then stamped onto the substrate surface. A cross-shaped tool (Figure 2d) showed consistent results with regard to epoxy adhesive volume and its homogeneous distribution on the substrate. The heated vacuum tool detached a chip from the thermal release foil and placed the chip on a flat surface to cool down. The tool was exchanged from a heated tool to a cold placement tool at the tool change station (Figure 4). The cold tool picked up the cooled chip from the storage area. The position and orientation of the chip vacuumed on the placement tool was controlled optically by image recognition of a bottom camera (Figure 3f). This allowed for a compensation of lateral and rotational offset during the placement step after picking the chip off the cooling stage (Figure 3e). A careful placement of the chip onto the epoxy adhesive was realized in three height steps for controlled spreading of epoxy adhesive. At the first step the chip was moved downwards until 200 µm above the substrate for a first contact with the epoxy adhesive. Further, the chip was moved 100 µm/s downwards until 100 µm above the substrate to allow homogeneous distribution of the epoxy adhesive. The remaining 100 µm were moved with the machines’ minimum traverse speed of 20 µm/s until touchdown of the chip on the substrate. The tool was left in touchdown position for 30 s with activated vacuum. Afterwards, the tool vacuum was stopped, finishing the placement process. Using this described process, nine ultrathin chips were placed on epoxy adhesive on a glass substrate with a laser-scribed grid. After placement, the glass substrate was taken out of the assembly machine and placed in an oven at 80 °C for two hours to cure all epoxy adhesive bonds simultaneously, similarly to the procedure with a single chip. It was observed that the chips floated on the liquid epoxy adhesive before the adhesive was cured and solid. This led to misalignment due to missing fixation, as seen in Figure 5. 

**Figure 6:**
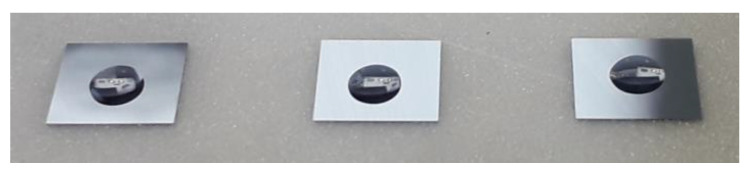
Epoxy adhesive is dispensed onto the backside of the chips after automated face-down placement on thermal release foil.

To eliminate movement of the chips during curing, the ultrathin chips were placed face-down on adhesive thermal release foil with a cold placement tool using an automated placement process. The foil was fixated onto a substrate holder using adhesive tape. After face-down placement of chips on the foil, the foil was taken out of the machine and epoxy adhesive (EPO-TEK 354, Epoxy Technology, Billerica, Massachusetts, USA) was dispensed onto the backside of the chips (Figure 6). 

The target substrate, e.g., glass or polyimide foil, was put on top of the chips and the epoxy adhesive. Excessive adhesive was squeezed out of the bonding layer by applying pressure onto the bonding location. The epoxy adhesive was cured at 80 °C for two hours in an oven while the thermal release foil fixated the chips and the target substrate mechanically. After curing, the temperature was increased to 120 °C to reduce the adhesive force of the thermal release foil. The thermal release foil was peeled off and the ultrathin chips remained adhesively bonded on the substrate. The thickness as well as the adhesive distribution of the adhesive underneath the chips on the glass substrate was measured using white light interferometry (Wyko NT 9100, Bruker, Billerica, MA, USA).

### 2.3. Measurement of Chip Placement Accuracy

**Figure 7:**
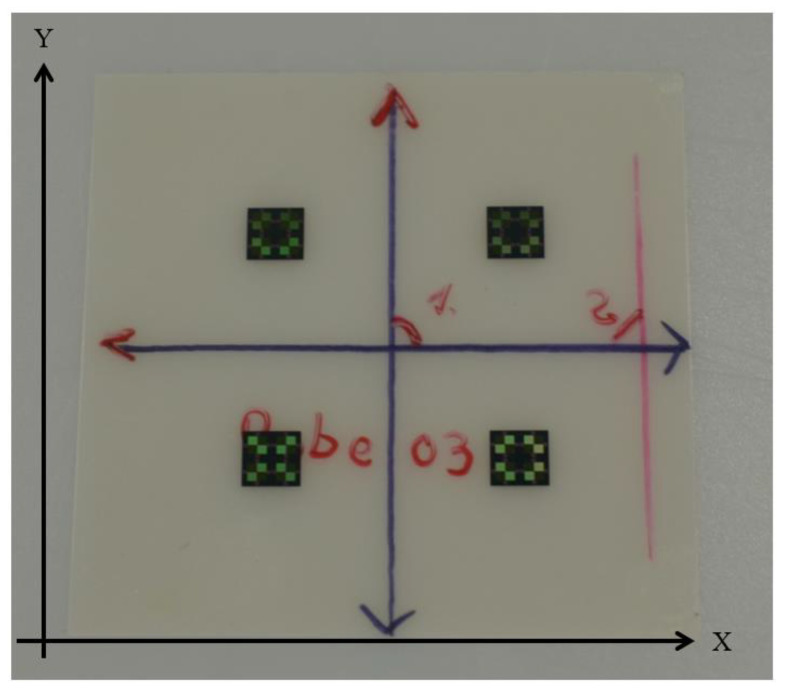
Four ultrathin chips placed on thermal release foil. Before placing, a coordinate cross was sketched on the foil creating a point of origin. The nominal distance of each chip from the origin is 10 mm in x- and y-orientation.

A coordinate system was established on a thermal release foil before placement to create a point of origin in the center of the foil, which was also set as the origin in the coordinate systems of the automatic assembly machine and the video measurement system (iNEXIV VMA-2520, Nikon, Tokyo, Japan). Four chips were placed face-up onto thermal release foil in a quadratic layout with a nominal relative distance of 10 mm in x- and y-orientation from the origin (Figure 7). In contrast to the automated face-down placement process described in Section 2, subheading B, the chips were placed face-up for the measurement of chip placement accuracy. The chips featured four symmetrically arranged Wheatstone bridges, which were clearly identifiable in the video measurement system. These features were used for the determination of placement accuracy. 

**Figure 8:**
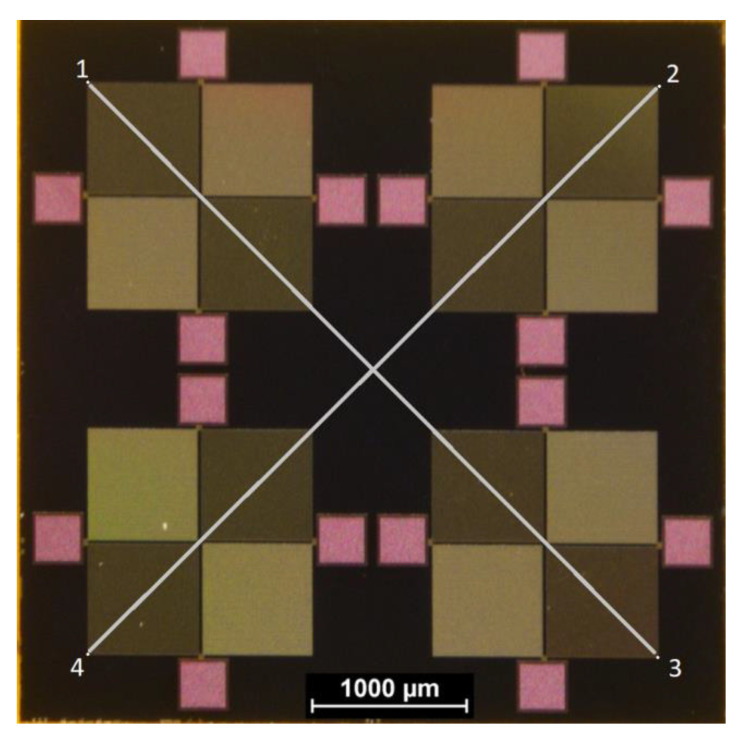
The surface of the assembled chips shows four Wheatstone bridges, designed in a symmetric layout. For the measurement of placement tolerance, four virtual points were defined for each Wheatstone bridge. Opposite points were virtually connected by lines and their interconnection marks the center of the chip.

One virtual point was set per Wheatstone structure. For the determination of the chip’s center point, opposite virtual points were connected by virtual lines, as seen in Figure 8. Thereby, the intersection point of the virtual lines defined the position of each chip in the initially set coordinate system on the thermal release foil. These positions were used to calculate the relative placement tolerance between two chips. The rotational misalignment was determined, calculating the angle between the virtual line of the two lower virtual Points 3 and 4 in Figure 8 on a chip and the line between the coordinate system origin and a second reference point on the x-axis of the coordinate system near the edge of the foil. 

## 3. Results

**Figure 9:**
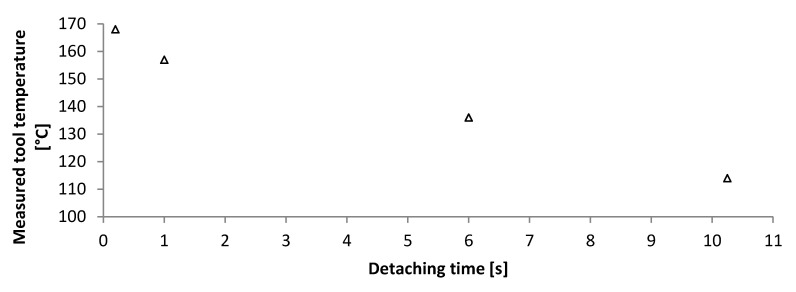
Relation between measured tool temperature and detachment time of ultrathin chips from thermal release foil. Temperatures higher than 160 °C ensure quick detachment.

It was found that a temperature of 120 °C reduced the adhesion force of the wafer dicing tape onto the ultrathin chips sufficiently for chip detachment. The surrounding chips were not influenced. Increasing the temperature above 160 °C reduced the detachment time to less than a second (Figure 9). 

An important finding was the need for a homogeneous vacuum area. An inhomogeneous vacuum area leads to warpage of the ultrathin chips while being handled. Besides the risk of breakage of the ultrathin components, the warpage prevents a homogeneous bonding layer thickness during the adhesive-based die attachment process. Tools with a uniform distribution of vacuum channels and smaller area (4.5 mm × 4.5 mm) than the used chip size (4.7 mm × 4.7 mm) were successfully utilized without damages to the chips. 

**Figure 10:**
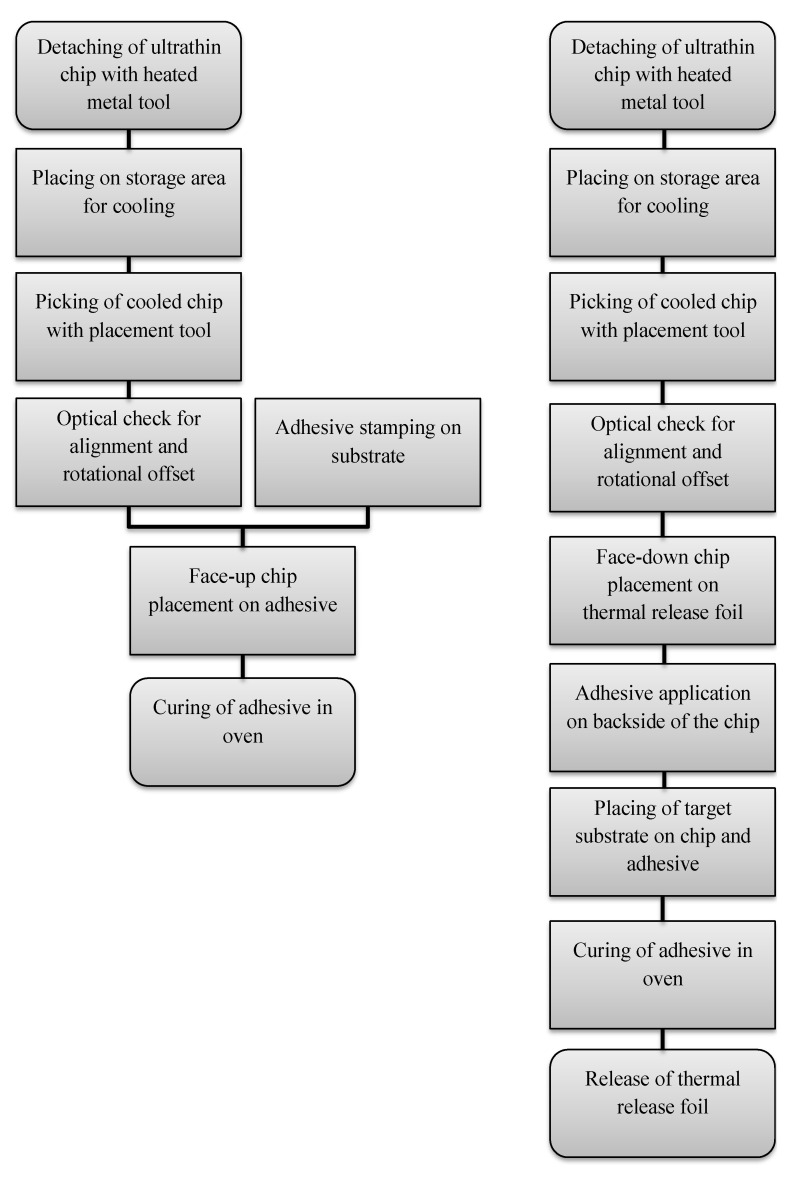
Process flow chart for automated face-up placement of ultrathin chips on stamped epoxy adhesive on the target substrate (**left**) and automated face-down placement of ultrathin chips on thermal release foil and subsequent application of epoxy adhesive on the backside of the chip (**right**).

**Figure 11:**
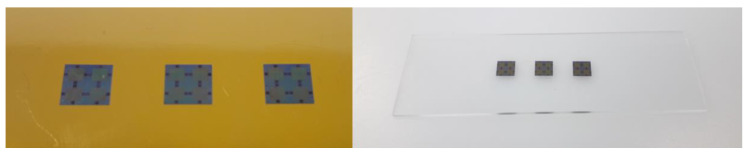
Adhesively bonded ultrathin chips on polyimide foil (**left**) and glass substrate (**right**).

The automated process included the successful detachment of ultrathin chips, stamping of adhesive, and the placement of ultrathin chips on adhesive. Two separated metal tools were used, a heated tool and a cold tool, because tools in this automatic assembly machine could only be actively heated, but not actively cooled. There is the risk of contamination with adhesive of already assembled components if only a heated tool is used for the placement of the chips. Liquid adhesive can squirt out of the adhesive gap due to the decreasing viscosity of the adhesive by increased temperature before curing and the simultaneously rapid expansion of trapped air within the adhesive. If multiple chips are simultaneously cured in an oven, the ultrathin chips need to be mechanically fixated during adhesive curing to avoid misalignment due to floating on liquid adhesive. For this purpose, the chips were automatically placed on adhesive thermal release foil and transferred onto a target substrate using epoxy adhesive. Thereby, adhesive thermal release foil covered the chip surface during the placement of the target substrate on the adhesive. The process flow using the placement of ultrathin chips on epoxy adhesive, as well as the process flow of the placement of ultrathin chips on adhesive thermal release foil, and the subsequent transfer to a target substrate are visualized in Figure 10. Figure 11 shows the successfully conducted process for the target substrates polyimide foil and glass. 

**Figure 12:**
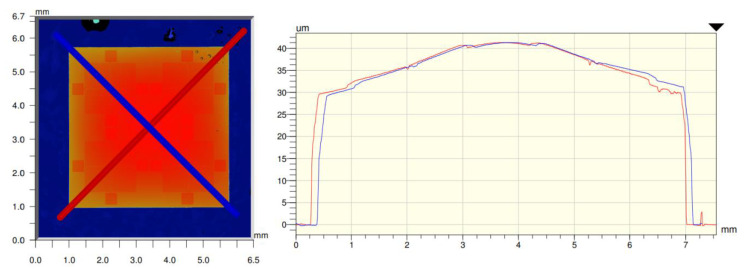
White light interferometry of an adhesively bonded 30-µm ultrathin silicon chip on glass substrate (**left**). The blue and red diagonals on the left side refer to the blue and red graphs on the right side, respectively (**right**).

The adhesive thickness measurements showed adhesive thickness of maximum 10 µm in the center of the chip (Figure 12) similar to [30]. The adhesive accumulated in the center, resulting in a decreasing adhesive thickness towards the edge of the chip. The gradient angle from the edge to the center resulted in 0.24°. During the placement of the target substrate onto the liquid adhesive, excessive adhesive was spread around the covered chip and remained on the target substrate after curing. The measurement of relative placement tolerance for ultrathin chips resulted in ±25 µm in x- and y-orientation. The calculation of rotational misalignment resulted in maximal 0.15°. 

## 4. Discussion

Silicon chips become mechanically flexible if thinned down below 50 µm thickness. However, silicon is a brittle material. Therefore, one important factor of this research was the detachment process of ultrathin chips with thicknesses of 30 µm from thermal release foil and their placement on a substrate. In the beginning, the detachment process was investigated. The choice of a suitable thermal release foil has to be made in regard to required adhesion strength and vacuum strength of the detachment tool as well as thermal stability of involved materials and components. If the vacuum strength is too low, the chips cannot be detached due to too high adhesion strength of the thermal release foil. If the vacuum strength is too high, the chips could be damaged during the detachment process. 

Further, it was found that the detachment tools must provide a homogenous vacuum area to avoid warpage of the flexible chips. A placing of warped chips onto liquid adhesive can lead to an uneven distribution or local accumulation of adhesive below the chip after adhesive curing. A smaller tool area than the chip size avoided contamination of the chip surface with adhesives by capillary forces [17]. Even small contaminations of the tool or the components are critical because cured adhesive on the chip contact pads will prevent contacting the chips in later process steps and the tool can become unusable if contaminated with cured adhesive.

The placement of ultrathin chips on stamped adhesive on the target is possible, but requires curing of the adhesive while the chip is fixated at the placement tool. This procedure can result in long production times for substrates with many components, depending on the used adhesive system. The assembly time can be reduced if an assembly machine with placement tool with active cooling system or an adhesive system with rapid curing is used. The risk of contamination of the placement tool with liquid adhesive during assembly remains.

The placement of chips on adhesive thermal release foil and the transfer onto a target substrate can reduce the time for adhesive curing because all adhesive bonds can be cured simultaneously. Furthermore, the separation of chip placement and adhesive process eliminates the risk of adhesive contamination of the placement tool. Moreover, the possibility to use different adhesive systems allows for various target materials besides the tested polyimide and glass. After optimization of the detachment process and after the separation of automated placing process and adhesive curing, all detached chips were placed and bonded successfully without damages to the chips or to the tools. 

The resulting relative placement accuracy of ±25 µm, rotational misalignment of 0.15°, and the homogeneous adhesive thickness of maximum 10 µm allowed for subsequent photolithographic processes after embedding the chips in photosensitive resist [31]. Electrical contacting could be done with physical vapor deposition (PVD), chemical vapor deposition (CVD), or digital printing technologies using inks with metallic nanoparticles.

## 5. Conclusions

This paper demonstrated the feasibility of an automated process for ultrathin chip assembly utilizing standard automatic assembly machines. Special tools were manufactured that allowed detachment of ultrathin chips with 30 µm thickness without die-ejector tools within seconds. It was possible to automate the handling of ultrathin chips and placing on liquid adhesive as well as on adhesive foil. To achieve high placement accuracy, the chips had to be mechanically fixated during adhesive curing to avoid floating. The method of separating the process steps of chip placement and the curing of adhesive offered high relative placement accuracy and short processing times. The resulting relative placement accuracy of ±25 µm, rotational misalignment of 0.15°, and the homogeneous adhesive thickness of maximum 10 µm allowed for subsequent photolithographic processes. To conclude, it is feasible to automate the detachment and placing of ultrathin chips using standard automatic assembly machines. 

## References

[1] Koyuncu M., Lorenz E., Zimmermann A., Logothetidis S. (2014). Advanced interconnection technologies for flexible organic electronic systems. Handbook of Flexible Organic Electronics; Materials, Manufacturing and Applications.

[2] Gupta S., Navaraj T.W., Lorenzelli L., Dahiya R. (2018). Ultra-thin chips for high-performance flexible electronics. NPJ Flex. Electron..

[3] Klink G., Feil M., Ansorge F., Reichl H. Assembly of ultra thin and flexible ICs. Proceedings of the 4th International Conference on Adhesive Joining and Coating Technology in Electronics Manufacturing.

[4] Takyu S., Kurosawa T., Shimizu N., Harada S. Novel Wafer Dicing and Chip Thinning Technologies Realizing High Chip Strength. Proceedings of the 56th Electronic Components and Technology Conference 2006.

[5] Zimmermann M., Burghartz J.N., Apple W., Remmers N., Burwick C., Wurz R., Tobail O., Schubert M., Palfinger G., Werner J. A seamless ultra-thin chip fabrication and assembly process. Proceedings of the 2006 International Electron Devices Meeting.

[6] Dahiya R.S., Adami A., Collini C., Lorenzelli L. Bendable Ultra-Thin Silicon Chips on Foil. Proceedings of the SENSORS, 2012 IEEE.

[7] Yoshikawa K., Miyazaki T., Watanabe N., Aoyagic M. (2012). Wet-Chemical Silicon Wafer Thinning Process for High Chip Strength. ECS. Trans..

[8] Angelopoulos E.A., Zimmermann M., Appel W., Endler S., Ferwana S., Harendt C., Hoang T., Pruemm A., Burghartz J.N. Ultra-thin chip technology for system-in-foil applications. Proceedings of the 2010 International Electron Devices Meeting.

[9] Van den Brand J., Kusters R., Barink M., Dietzel A. (2010). Microelectronic Engineering Flexible embedded circuitry: A novel process for high density, cost effective electronics. Microelectron. Eng..

[10] Haberland J., Becker M., Kallmayer C., Aschenbrenner R., Reichl H. Ultrathin 3D ACA FlipChip-in-Flex Technology. Proceedings of the International Conference and Exhibition on Device Packaging.

[11] Van Den Brand J., Kusters R., Heeren M., Van Remoortere B., Dietzel A. Flipchip bonding of ultrahin Si dies onto PEN/PET substrates with low cost circuitry. Proceedings of the 3rd Electronics System Integration Technology Conference ESTC.

[12] Christiaens W., Loeher T., Pahl B., Feil M., Vandevelde B., Vanfleteren J. (2008). Embedding and assembly of ultrathin chips in multilayer flex boards. Circuit World.

[13] Alavi G., Sailer H., Albrecht B., Harendt C., Burghartz J.N. (2018). Adaptive Layout Technique for Microhybrid Integration of Chip-Film Patch. IEEE Trans. Compon. Packag. Manuf. Technol..

[14] Sridhar A., Cauwe M., Fledderus H., Kusters R.H.L., van den Brand J. Novel interconnect methodologies for ultra-thin chips on foils. Proceedings of the 2012 IEEE 62nd Electronic Components and Technology Conference.

[15] Bock K., Yacoub-George E., Hell W., Drost A., Wolf H., Bollmann D., Landesberger C., Klink G., Gieser H., Kutter C. Multifunctional System Integration in Flexible Substrates. Proceedings of the IEEE 64th Electronic Components and Technology Conference (ECTC).

[16] Hassan M.-U., Schomburg C., Penteker E., Harendt C., Hoang T., Burghartz J.N. Imbedding Ultra-Thin Chips in Polymers. Proceedings of the Dutch Conference ICT.OPEN.

[17] Harendt C., Kostelnik J., Kugler A., Lorenz E., Saller S., Schreivogel A., Yu Z., Burghartz J.N. (2015). Hybrid Systems in Foil (HySiF) exploiting ultra-thin flexible chips. Solid State Electron..

[18] Wolf J., Kostelnik J., Berschauer K., Kugler A., Lorenz E., Harendt C., Yu Z. Ultra-thin Silicon Chips in Flexible Microsystems. Proceedings of the ECWC 13, 13th Electronic Circuits World Convention.

[19] Burghartz J.N., Rempp W.A., Zimmermann M. (2009). A New Fabrication and Assembly Process for Ultrathin Chips. IEEE Trans. Electron Devices.

[20] Hassan M., Schomburg C., Harendt C., Penteker E., Burghartz J.N. Assembly and Embedding of Ultra-Thin Chips in Polymers. Proceedings of the 2013 Eurpoean Microelectronics Packaging Conference (EMPC).

[21] Kuo T.-Y., Shih Y.-C., Lee Y.-C., Chang H.-H., Hsiao Z.-C., Chiang C.-W., Li S.-M., Hwang Y.-J., Ko C.-T., Chen Y.-H. Flexible and ultra-thin embedded chip package. Proceedings of the 2009 59th Electronic Components and Technology Conference.

[22] Wang L., Sterken T., Cauwe M., Cuypers D., Vanfleteren J. (2012). Fabrication and Characterization of Flexible Ultrathin Chip Package Using Photosensitive Polyimide. IEEE Trans. Compon. Packag. Manuf. Technol..

[23] Xue X., Yang S., Wu D., Pan L., Wang Z. Fabrication of ultra-thin silicon chips using thermally decomposable temporary bonding adhesive. Proceedings of the 2016 IEEE Sensors.

[24] Manessis D., Boettcher L., Karaszkiewicz S., Ostmann A., Aschenbrenner R., Lang K.-D. Chip embedding technology developments leading to the emergence of miniaturized system-in-packages. Proceedings of the 18th European Microelectronics & Packaging Conference.

[25] Govaerts J., Christiaens W., Bosman E., Vanfleteren J. (2009). Fabrication Processes for Embedding Thin Chips in Flat Flexible Substrates. IEEE Trans. Adv. Packag..

[26] Christiaens W., Torfs T., Huwel W., Van Hoof C., Vanfleteren J. 3D integration of ultra-thin functional devices inside standard multilayer flex laminates. Proceedings of the 2009 European Microelectronics and Packaging Conference.

[27] Van den Brand J., de Baets J., van Mol T., Dietzel A. (2008). Systems-in-foil—Devices, fabrication processes and reliability issues. Microelectron. Reliab..

[28] Liu Z., Huang Y.A., Liu H., Chen J., Yin Z. (2014). Reliable Peeling of Ultrathin Die With Multineedle Ejector. IEEE Trans. Compon. Packag. Manuf. Technol..

[29] Feil M., Landesberger C., Bock K. The challenge of ultra thin chip assembly. Proceedings of the 54th Electronic Components and Technology Conference.

[30] Yang S., Zhao P., Song Z., Wang Z. The flexible package and applications of ultra-thin sensor chip. Proceedings of the 2015 16th International Conference on Electronic Packaging Technology (ICEPT).

[31] Janek F., Weser S., Barth M., Eberhardt W., Zimmermann A. (2019). Assembly of Multiple Ultrathin Chips on Flexible Foils with High Placement Accuracy by a Simple Transfer Process. IEEE Trans. Compon. Packag. Manuf. Technol..

